# Compartmentalization of HIV-1 within the Female Genital Tract Is Due to Monotypic and Low-Diversity Variants Not Distinct Viral Populations

**DOI:** 10.1371/journal.pone.0007122

**Published:** 2009-09-22

**Authors:** Marta Bull, Gerald Learn, Indira Genowati, Jennifer McKernan, Jane Hitti, David Lockhart, Kenneth Tapia, Sarah Holte, Joan Dragavon, Robert Coombs, James Mullins, Lisa Frenkel

**Affiliations:** 1 Seattle Children's Hospital Research Institute, Seattle, Washington, United States of America; 2 University of Washington, Seattle, Washington, United States of America; 3 Fred Hutchinson Cancer Research Center, Seattle, Washington, United States of America; University of California San Francisco, United States of America

## Abstract

**Background:**

Compartmentalization of HIV-1 between the genital tract and blood was noted in half of 57 women included in 12 studies primarily using cell-free virus. To further understand differences between genital tract and blood viruses of women with chronic HIV-1 infection cell-free and cell-associated virus populations were sequenced from these tissues, reasoning that integrated viral DNA includes variants archived from earlier in infection, and provides a greater array of genotypes for comparisons.

**Methodology/Principal Findings:**

Multiple sequences from single-genome-amplification of HIV-1 RNA and DNA from the genital tract and blood of each woman were compared in a cross-sectional study. Maximum likelihood phylogenies were evaluated for evidence of compartmentalization using four statistical tests. Genital tract and blood HIV-1 appears compartmentalized in 7/13 women by ≥2 statistical analyses. These subjects' phylograms were characterized by low diversity genital-specific viral clades interspersed between clades containing both genital and blood sequences. Many of the genital-specific clades contained monotypic HIV-1 sequences. In 2/7 women, HIV-1 populations were significantly compartmentalized across all four statistical tests; both had low diversity genital tract-only clades. Collapsing monotypic variants into a single sequence diminished the prevalence and extent of compartmentalization. Viral sequences did not demonstrate tissue-specific signature amino acid residues, differential immune selection, or co-receptor usage.

**Conclusions/Significance:**

In women with chronic HIV-1 infection multiple identical sequences suggest proliferation of HIV-1-infected cells, and low diversity tissue-specific phylogenetic clades are consistent with bursts of viral replication. These monotypic and tissue-specific viruses provide statistical support for compartmentalization of HIV-1 between the female genital tract and blood. However, the intermingling of these clades with clades comprised of both genital and blood sequences and the absence of tissue-specific genetic features suggests compartmentalization between blood and genital tract may be due to viral replication and proliferation of infected cells, and questions whether HIV-1 in the female genital tract is distinct from blood.

## Introduction

Distinct genetic populations of HIV-1 in the genital tract compared to blood have been reported in 170 men [1], [2], [3], [4], [5] and 57 women [6], [7], [8], [9], [10], [11], [12], [13], [14], [15], [16], [17], [18]. Physical partitions, cellular membranes, inadequate penetration of antiretroviral drugs into the genital tract [19], [20] or localized inflammation [7], [8], [9] are hypothesized to facilitate replication in the genital tract independently from the blood, and allow evolution of HIV-1 genital tract variants that appear distinct from the blood [6], [7], [8].

The central role of the female genital tract in both sexual and perinatal HIV-1 transmission underscores the importance of understanding viral evolution within these tissues, which should contribute to the development of effective treatments and prevention strategies, including vaccines. While multiple reports suggest that HIV-1 appears compartmentalized between the genital tract and the blood of women [6], [7], [8], [9], [10], [11], [12], [13], [14], [15], [16], [17], [18], [21], we hypothesized that the absence of strict physical barriers between the female genital tract and blood would allow viruses to mix between these two tissues.

Studies reporting compartmentalization of viruses within the female genital tract have often analyzed only cell-free viruses [8], [9], [11], [12], [13], [15], [16], [17], which, given the rapid turnover of HIV-1, are derived primarily from recent cycles of replication. We expand on these studies by analyzing both cell-free HIV-1 RNA and cell-associated HIV-1 DNA to sample both replicating and archived viruses, respectively, in the blood and genital tract. Our sequencing of single genome-amplifications of a relatively large sampling of viruses from these two tissues allowed us to carefully consider whether HIV-1 is compartmentalized between the genital tract and blood of women.

## Materials and Methods

### Study design

A cross-sectional study of chronically HIV-1 infected women compared viral populations in each woman's genital tract to her blood. Viral sequences were derived by single genome amplification (SGA). To control for variations in HIV-1 shedding throughout the menstrual cycle subjects' study visits were conducted during the luteal phase of their cycle [22]. Demographic, medical, and reproductive health information was collected from medical records. The study was conducted at University of Washington, following procedures approved by the Institutional Review Board, and after subjects' written consent.

Study eligibility was not limited by use of antiretroviral drugs. Participants were classified into one of three categories according to antiretroviral treatment (ART) and plasma viral load at the time specimens were collected: “effective ART”, when virus replication was suppressed to <50 copies/mL; “failing ART”, defined as receiving ART, but with plasma HIV-1 RNA >400 c/mL; and “no ART”, when not receiving ART.

### Ethics Statement

The study was conducted at University of Washington, following procedures approved by the University of Washington Institutional Review Board. All study participants provided written informed consent.

### Specimen processing

Blood plasma and PBMC were separated using Accuspin™ tubes (Sigma-Aldrich, St. Louis, MO). Cells were counted using a Beckman Coulter Z1 Coulter Particle Counter (Brea, CA) or hemocytometer. The cell pellet and plasma fraction were stored at −80°C until nucleic acids were extracted.

Cervical secretions were obtained by placing three filter paper wicks (Sno-Strip™, Chauvin Pharmaceuticals, Romford, Essex, UK) into the cervical *os*. After absorption of ∼24 uL of fluid the strips were visually inspected for blood contamination and placed in guanidinium isothiocyanate extraction solution (4 M; Sigma-Aldrich) [22]. After application of lidocaine spray, a single-punch biopsy of the cervix adjacent to the *os* was obtained using Baby Tischler Cervical Biopsy Forceps (Howard Medical Company, Chicago, IL) and placed into RPMI. The cervical tissues were snap-frozen in Optimal Cutting Temperature freezing medium (Tissue-Tek, VWR, West Chester, PA) by floating in a cryomold (Tissue-Tek) on top of 2-methyl-butane cooled by dry ice. Tissues were stored at −80°C until sectioned for DNA extraction.

### Quantification of HIV-1 RNA

Virions in 500 µL of plasma were concentrated by ultra-centrifugation. Guanidinium isothiocyanate extraction solution (4 M; Sigma-Aldrich) was added to viral pellet. GeneAmplimer pAW 109 RNA plasmid (Applied Biosystems, Foster City, CA) was added to plasma virus or 300 µL of cervical secretions in guanidinium solution. Silica was used to extract nucleic acids [23]. Extracted RNA was reverse transcribed and quantified in a one-step RT-PCR real-time assay (Roche Diagnostics, Indianapolis, IN and ABI Prism 7700, Foster City, CA) as described [24], [25].

### Quantification of cell-associated HIV-1 DNA

DNA was extracted from PBMC using the IsoQuick nucleic acid extraction kit (Orca Research Inc., Bothell, WA). Cervical DNA was isolated from 4 to 8 sections, each 20 µM thick, of the punch biopsy tissue, using the Gentra DNA Purification System (Minneapolis, MN). Viral genome of extracted DNA and some cDNA samples were quantified using limiting dilution PCR [26], [27].

### Sequencing of multiple HIV-1 RNA and DNA genomes by single-genome-amplification (SGA)

The nucleic acids in 0.2–1 mL of plasma and 20–50 µL of genital secretions were extracted using either the Qiagen QIAamp Viral RNA Mini-kit (Qiagen Inc., Valencia, CA) or when the viral load was below the limit of detection (<50 copies/mL) using silica with slight modifications [23]. Purified RNA was reverse transcribed using Superscript II (Invitrogen, Carlsbad, CA) into cDNA with BH2 primer for *env* [28].To control for amplification of cell associated HIV-1 DNA in plasma and genital Sno-strip samples, RNA extracted from specimens were pooled across multiple subjects into a “no reverse transcriptase (RT)” control, and PCR for HIV-1 *env* was conducted as described below. None of these controls yielded amplicons, suggesting negligible DNA contamination in plasma and Sno-strip specimens from these subjects. Viral gene sequences corresponding to the C2-V5 region of HIV-1 *env* were derived as previously described [24]. This region of *env* was chosen for its genetic diversity and because it amplifies in multiplexed end-point dilution PCR at a sensitivity similar to regions encoding RT and PR [27].

### Sequence analysis and phylogram construction

Sequences were assembled and checked for read errors and hypermutation [27]. An all-inclusive phylogram was constructed to verify that each sequence segregated only with others from the identified participant. An evolutionary model was selected in PAUP* version 4.0b10 using Modeltest version 3.7 and the Akaike information criterion as previously described [27]. A phylogenetic tree based on maximum likelihood estimation for each subject's sequences was generated using PAUP as previously described [24].

### Evaluation of peripheral blood and genital tract sequences for evidence of viral compartmentalization

The compartmental structure of viral sequences from each subject was evaluated by four distinct statistical tests using either tree- or distance-based parameters: (1) Slatkin and Maddison (SM) evaluates variation from normalcy in the distribution of sequences over a predicted tree structure using MacClade [7], [29], [30]; (2) Hudson's nearest neighbor (S_nn_) compares genetic distances between sequences within and between tissues independent of phylogeny [31]; (3) The Critchlow correlation coefficient r_b_ compares the number of nodes between sequences within and between tissues; and (4) the Critchlow coefficient r compares tree-based genetic distances within and between tissues [32]. The SM test was further evaluated using 100 bootstrap replicate phylogenies [7], [33]. The S_nn_ and correlation coefficients were calculated in HyPhy with 1,000 permutations between tissues to determine statistical significance [34], [35]. P-values of <0.0125 were considered to be evidence of compartmentalization after applying a Bonferroni correction. The topology of each phylogenetic tree was also reviewed, with particular attention to genital tract or peripheral blood specific clades.

### Analysis of HIV-1 diversity and divergence

Divergence of each sequence from the MRCA and population diversity (estimated as the average pair-wise genetic distances) were calculated using PAUP* version 4.0b10 with the corresponding evolutionary model. The mean diversity and divergence were calculated for each subject's anatomical sites (plasma RNA, PBMC DNA, genital RNA, and genital DNA).

### Analysis of amino acid variation in HIV-1 *env*

Non-synonymous and synonymous distances (dN and dS, respectively) and dN/dS ratios were based on maximum likelihood trees with codon substitution models (codeml, PAML version 3.15) [36], [37]. The NSsites Model 0 was used to estimate the dN/dS ratio for a set of sequences. Evidence of positive selection is indicated by a dN/dS score >1. Selection at amino acid sites was also ascertained using codeml, but with NSsites model 8, by Bayes empirical Bayes (BEB) analysis for positive selection with posterior probability >95% [38].

Atypical amino acid residues in genital tract sequences compared with peripheral blood sequences were evaluated using the aligned protein sequences from the C2-V5 *env* region in the Viral Epidemiology Signature Pattern Analysis program (http://www.hiv.lanl.gov/content/hiv-db/P-vespa/vespa.html) [39]. Shannon entropy scores [40] were calculated for each position in the protein alignment using the Entropy2 program (http://hiv-web.lanl.gov/content/hiv-db/ENTROPY/entropy.html).

The number of potential N-linked glycosylation sites in blood and genital tract sequences was determined using the N-GLYCOSITE program [41] (http://hiv-web.lanl.gov/content/hiv-db/GLYCOSITE/glycosite.html). HIV-1 co-receptor usage was predicted for the V3 region amino acid sequences using the position-specific scoring X4/R5 and SI/NSI matrices (subtype B sequences), and/or the presence of basic amino acids at V3 region sites 11 or 25 (all sequences) [42] (http://indra.mullins.microbiol.washington.edu/pssm/).

### Statistical analysis

Confidence intervals for entropy, average dN/dS values, and mean diversity values were calculated using JMP (JMP, SAS Institute, Cary, NC). Viral divergence values, number of dN/dS sites and entropy between sequence sources (plasma, PBMC, cervical RNA, and cervical DNA) were compared using Wilcoxon Rank Sums tests (JMP, Cary, NC). Diversity values were calculated using the mean pair-wise distances for all sequence comparisons from a tissue [43]. Agreement between tests, the SM, S_nn_, and Critchlow correlations, was evaluated using GraphPad software and expressed as a κ−score (http://www.graphpad.com/quickcalcs/CatMenu.cfm).

DNA sequences derived from biopsied cervical tissue could be from infected genital tract cells or from cells circulating in blood vessels within the cervical tissue. Pair-wise distances generated in PAUP were used to identify whether the nearest neighbor to each genital DNA sequence was another genital tract or a blood sequence. For each patient, generalized estimating equations (GEE) with an exchangeable correlation matrix was used to calculate 95% confidence intervals for the proportion of pair-wise comparisons where genital DNA sequences were nearest to other genital RNA and/or DNA sequences using SAS v9.1 (Cary, NC). Under the assumption of random matching, we calculated the random probability (proportion) that any sequence would be matched with a genital DNA or genital RNA sequence [(# genital RNA + # genital DNA sequences)/(# total sequences per patient)]. If the random proportion fell outside of the GEE-based confidence intervals, we concluded there was statistically significant evidence that genital DNA sequences were more likely from the cervix rather than a contaminant from blood passing through cervical blood vessels.

### Nucleotide sequence accession numbers

The gene sequences determined in this study were deposited in GenBank under accession numbers EF624488-EF625226.

## Results

### Analyses to determine compartmental structure of genital and blood HIV-1 populations

Cross-sectional analyses of HIV-1 populations in the genital tract and blood were conducted on thirteen subjects (Table 1). A median of 50 sequences was derived by SGA from each participant (Table 2). When the maximum likelihood phylograms from each subject's sequences were assessed for compartmentalization between genital and blood viruses the outcome varied across the four assays. Compartmentalization of genital viruses from the blood was detected by one or more statistical tests in 10/13 (77%), two or more in 7/13 (54%), three or more tests in 5/13 (38%), or by all four tests in 2/13 (15%) subjects (Table 3). As would be suggested by the poor concordance across these tests, the κ−score for correlations between the methods were low (0.09–0.40), except between the related Critchlow coefficients, r_b_ and r (κ score = 0.68).

**Table 1:** Clinical parameters of study participants.

Treatment Status	Subject	Years Post HIV-1 Diagnosis	Mode of HIV-1 Acquisition	Current ART	CD4 Cells/µL	CD4%	Plasma HIV-1 RNA c/mL	Cervical HIV-1 RNA c/mL
ART	1	12	Sexual	3TC, TDF, EFV	290	20	<50	<600^a^
	2	9	Sexual	3TC, d4T, NFV	30	3	<50	<600 a
“Failing” ART	3	4	Sexual	3TC, ZDV, EFV, NFV	280	32	35,000	1,500
	4	11	IDU/Sexual	3TC, d4T, EFV	160	19	6,000	2,100
	5	18	Sexual	3TC,ABC, NVP	ND	ND	16,700	<600
	6	10	Sexual	FTC, TDF, LPV/r	467	29	69,000	7,800
No ART	7	2	Sexual	None	640	38	11,000	1,500
	8	16	IDU/Sexual	None	460	21	16,000	18,000
	9	8	Sexual	None	909	10	100	15,000
	10	8	Sexual	None	366	23	50,000	<600
	11	1	Sexual	None	667	34	3,000	2,800
	12	8	Sexual	None	386	21	277,000	77,000
	13	1	Sexual	None	679	46	17,000	<600

Notes: Treatment status defined by whether receiving ART, and if so whether ART was “effective” with plasma levels <50 copies/mL, or “failing” defined as plasma HIV-1 RNA values of >400 c/mL after the initial 6 months of ART. FTC = Emtricitabine, 3TC = Lamivudine, d4T = Stavudine, ZDV = Zidovudine, EFV = Efavirenz, TDF = Tenofovir, LPV/r = Lopinavir/ritonavir, NFV = Nelfinavir, NVP = Nevirapine. IDU = Intravenous Drug Use, No ART = not receiving ARV, ND = No data.

a Limiting dilution PCR estimated HIV-1 RNA in these specimens between 150-35,000 copies/mL [26].

**Table 2:** The Number of Sequences Generated from Each Tissue examined in HIV-1 Infected Subjects.

Treatment status		Number of sequences analyzed			
	Subject ID	PBMC	Plasma	Cervical DNA	Cervical RNA
ART	1	18	3	14	6
	2	33	0	13	6
“Failing” ART	3	44	13	12	0
	4	40	10	11	5
	5	6	12	10	0
	6	16	10	10	11
No ART	7	30	10	17	1
	8	41	17	8	8
	9	14	11	13	0
	10	24	13	10	0
	11	30	9	13	9
	12	36	9	9	14
	13	19	11	10	0

Notes: Treatment status defined by whether receiving ART at time of study, and if so whether ART was “effective”, defined by plasma levels <50 HIV-1 RNA copies/mL, or “failing” defined as plasma HIV-1 RNA values of >400 c/mL after the initial 6 months of therapy.

**Table 3:** Evaluation of HIV-1 *env* for compartmentalization between the genital tract and blood using correlation coefficients, Slatkin-Maddison, and Nearest-Neighbor Models.

Treatment Status	Subject ID	Blood and Genital Tract (RNA and DNA) Evaluated	# of tests (+) for compartmentalization of HIV-1, with and without combining monotypic sequences
		r_b_ p-value^a^	r p-value^a^	S_nn_^b^ p-values	SM^c^ p-values	
“Effective” ART	1	<0.102	<0.049	0.011*	0.248	1
	2	<0.096	<0.008*	0.015	0.025	1
“Failing” ART	3	<0.145	<0.665	0.022	1.000	0
	4	<0.005*	<0.001*	0.005*	0.014	3
	4^d^	<0.041	<0.012*	0.067	0.492	1^d^
	5	<0.001*	<0.001*	<0.001*	<0.001*	4
	6	<0.789	<0.522	0.011*	0.047	1
No ART	7	<0.986	<0.515	0.062	0.045	0
	8	<0.009*	<0.001*	0.023	0.009*	3
	8^d^	<0.106	<0.001*	0.285	0.026	1^d^
	9	<0.035	<0.001*	<0.001*	<0.001*	3
	9^d^	<0.143	<0.001*	<0.001*	0.037	2^d^
	10	<0.106	<0.148	0.001*	<0.001*	2
	10^d^	<0.011*	<0.011*	0.009*	0.002*	4
	11	<0.133	<0.016	0.098	0.192	0
	12	<0.336	<0.036	<0.001*	0.002*	2
	13	<0.001*	<0.001*	0.010*	0.002*	4
	13^d^	<0.002*	<0.005*	<0.001*	0.220	3^d^
Total subjects # (%) with compartmentalization of genital and blood HIV-1 without collapsing		4 (31%)	6 (46%)	8 (62%)	6 (46%)	

Notes: Treatment status defined by whether receiving ART, and if so whether ART was “effective”, defined by plasma levels <50 copies/mL, or “failing” defined as plasma HIV-1 RNA values of >400 c/mL after the initial 6 months of therapy.

a–c Viral compartmentalization assessed by Critchlow correlation coefficients (r_b_ and r) [32], the nearest neighbor (S_nn_) [31] and Slatkin and Maddison (SM) [29].

d Evaluation of HIV-1 *env* compartmentalization between the genital tract and blood with identical sequences collapsed into a single sequence.

* Values p<0.0125 are considered significant after a Bonferroni correction for multiple comparisons.

**Figure 1:**
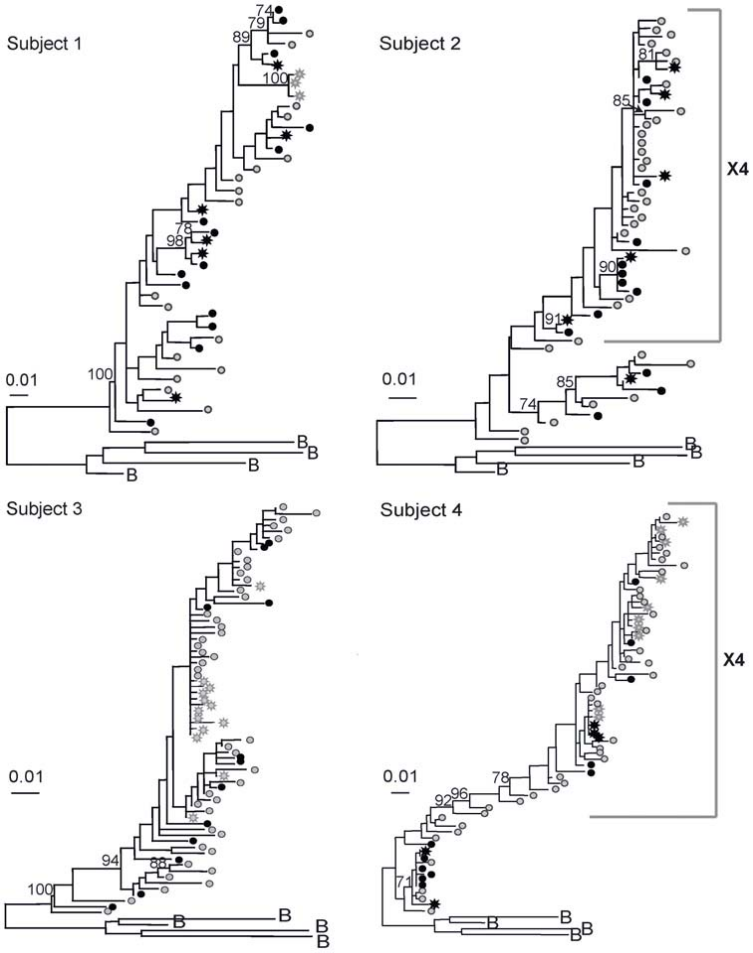
Maximum likelihood phylogenetic analyses of HIV-1 sequences corresponding to the C2-V5 region of *env*. Phylograms of blood and genital tract RNA and DNA sequences, derived by single-genome-amplification, are shown. Mixing of genital tract and blood sequences was noted in phylograms of most subjects. Subjects 1 and 2 were studied during effective ART (<50 copies/mL). Subjects 3 and 4 were studied during “failing” (>400 copies/mL) ART. HIV-1 sequences from plasma (gray stars); PBMC (gray circles), cell-free cervical RNA (black stars) and cell-associated cervical DNA (black circles) are shown. Sequences that were predicted to encode X4-tropic virus are indicated with brackets. Bootstrap values of >70% are indicated in each tree. Phylograms were rooted using representative sequences, indicated with the letter B, for the corresponding subtype from GenBank (Clade B: B.US.83.RF, B.US.90.WEAU160, B.FR.83.HXB2, B.US.86.JRFL). The scale bar (horizontal line) indicates the horizontal branch length corresponding to 1 substitution per 100 sites.

Close inspection of the five subjects with statistical evidence of compartmentalization by three or more tests revealed pairs and small clades of genetically similar and/or monotypic (identical) viruses from the genital tract and/or blood (Subjects 4, 5, 8, 9 and 13 in Figure 1–4), suggestive of a burst of viral replication or proliferation of infected cells. To explore whether recent replication or proliferation biased the statistical tests towards compartmentalization, identical sequences were collapsed to a single sequence and reanalyzed for compartmentalization. All five subjects had at least 10 sequences remaining from both the genital tract and the blood for this analysis. The re-assessment yielded significant p-values for fewer subjects (Table 3), showing that identical sequences inflate statistical measures of compartmentalization.

**Figure 2 pone-0007122-g002:**
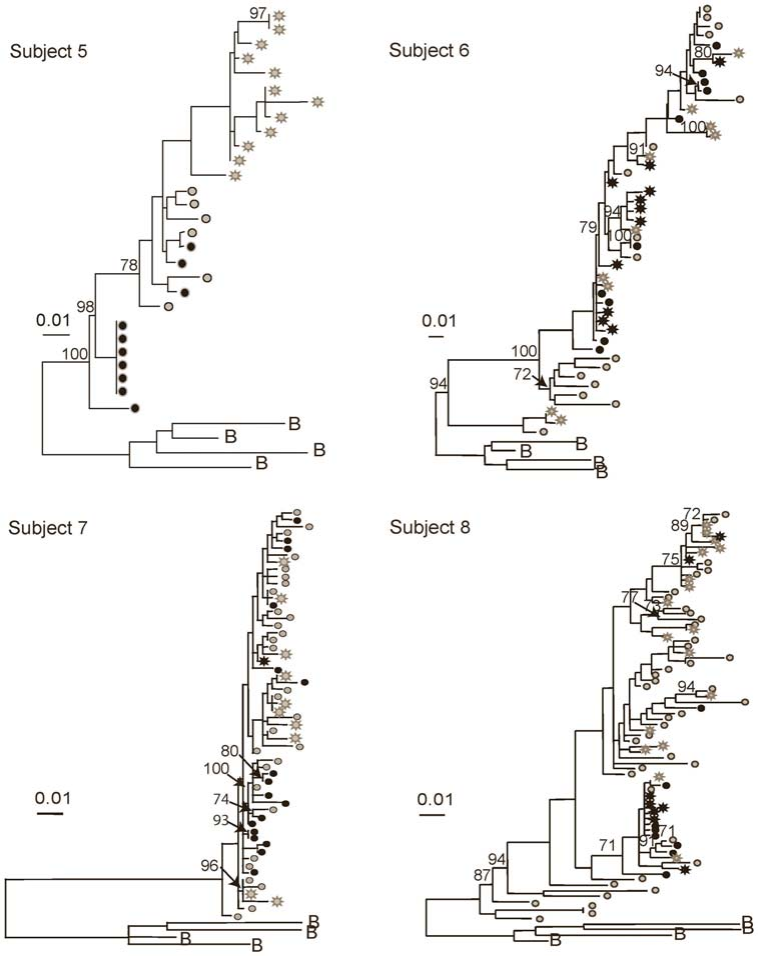
Maximum likelihood phylogenetic analyses of HIV-1 sequences corresponding to the C2-V5 region of *env*. Phylograms of blood and genital tract RNA and DNA sequences, derived by single-genome-amplification, are shown. Mixing of genital tract and blood sequences was noted in phylograms of most subjects. Subjects 5 and 6 were studied during “failing” (>400 copies/mL) ART. Subjects 7 and 8 were studied while not receiving ART. HIV-1 sequences from plasma (gray stars); PBMC (gray circles), cell-free cervical RNA (black stars) and cell-associated cervical DNA (black circles) are shown. Bootstrap values of >70% are indicated in each tree. Phylograms were rooted using representative sequences, indicated with the letter B, for the corresponding subtype from GenBank (Clade B: B.US.83.RF, B.US.90.WEAU160, B.FR.83.HXB2, B.US.86.JRFL). The scale bar (horizontal line) indicates the horizontal branch length corresponding to 1 substitution per 100 sites.

**Figure 3 pone-0007122-g003:**
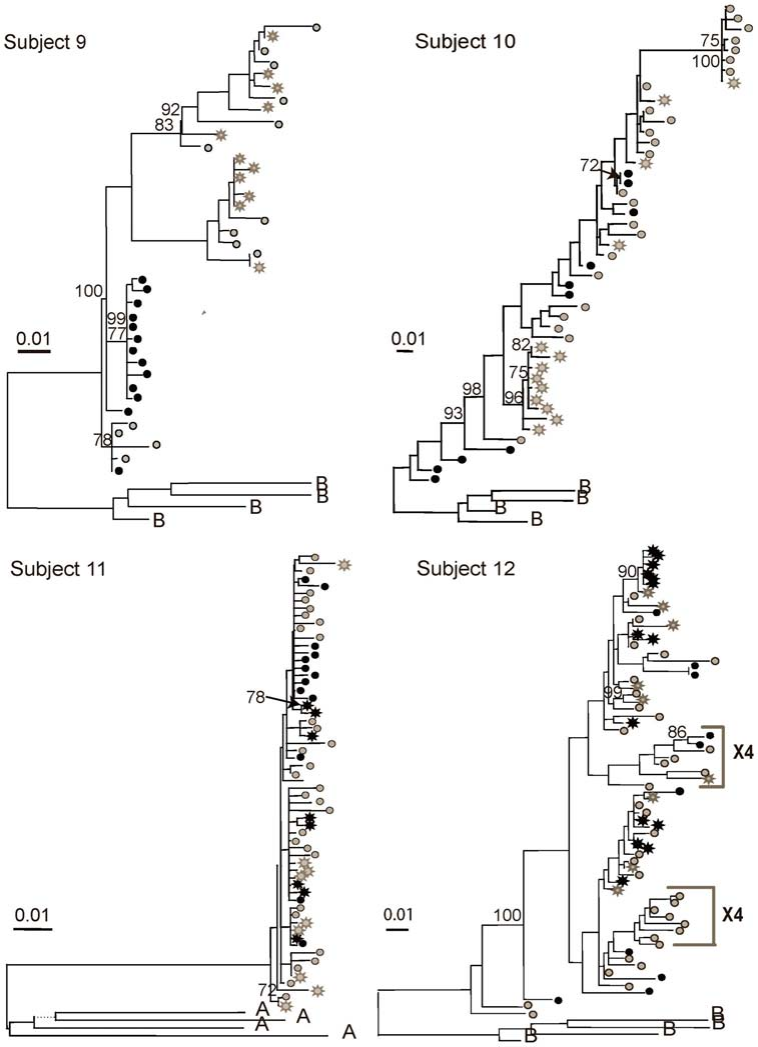
Maximum likelihood phylogenetic analyses of HIV-1 sequences corresponding to the C2-V5 region of *env*. Phylograms of blood and genital tract RNA and DNA sequences, derived by single-genome-amplification are shown. Mixing of genital tract and blood sequences was noted in phylograms of most subjects. Subjects 9, 10, 11, and 12 were studied while not receiving ART. HIV-1 sequences from plasma (gray stars); PBMC (gray circles), cell-free cervical RNA (black stars) and cell-associated cervical DNA (black circles) are shown. Sequences that were predicted to encode X4-tropic virus are indicated with brackets. Bootstrap values of >70% are indicated in each tree. Phylograms were rooted using representative sequences, indicated with the letter B or A, for the corresponding subtype from GenBank (Clade B: B.US.83.RF, B.US.90.WEAU160, B.FR.83.HXB2, B.US.86.JRFL; Clade A1 A1.KE.93.Q23-17, A1.SE.94.SE7253, A1.UG.92.92UG037, A1.UG.85.U455). The scale bar (horizontal line) indicates the horizontal branch length corresponding to 1 substitution per 100 sites.

**Figure 4:**
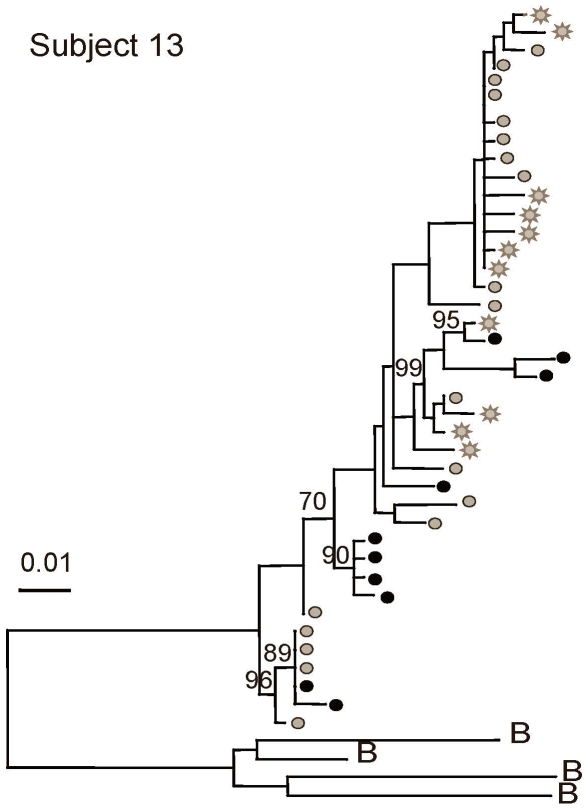
Maximum likelihood phylogenetic analyses of HIV-1 sequences corresponding to the C2-V5 region of *env*. Phylograms of blood and genital tract RNA and DNA sequences, derived by single-genome-amplification are shown. Mixing of genital tract and blood sequences were also noted in the phylogram of Subject 13 who was studied while not receiving ART. HIV-1 sequences from plasma (gray stars); PBMC (gray circles), cell-free cervical RNA (black stars) and cell-associated cervical DNA (black circles) are shown. Bootstrap values of >70% are indicated in each tree. Phylograms were rooted using representative sequences, indicated with the letter B, for the corresponding subtype from GenBank (Clade B: B.US.83.RF, B.US.90.WEAU160, B.FR.83.HXB2, B.US.86.JRFL). The scale bar (horizontal line) indicates the horizontal branch length corresponding to 1 substitution per 100 sites.

The three subjects with HIV-1 populations compartmentalized by three or more tests (Subjects 5, 9 and 13 Figure 2–4) had clades comprised of blood-only and genital- only sequences. The genital-only clades in each case included HIV-1 variants with low diversity, however, each subject also had one or more clades containing both blood and genital variants. Thus, despite statistically significant compartmentalization (Table 3), phylogram topologies showed intermingling of a portion of these subjects' genital and blood sequences (Figure 1–4).

Notably, across 11/13 subjects, phylogenetic clades with variants of ≥5% genetic distance, suggestive of an evolving viral lineage, included both genital and blood variants with the exception of Subjects 5 and 9. No unique clinical signs or laboratory parameters that might account for the genital tract clades of viruses were consistently identified in these two women (e.g. cervicitis, bacterial vaginosis, HSV shedding, elevation of inflammatory cytokines in vaginal lavage; data not shown) [44]. Each woman had a low diversity genital-specific clade and a large blood-specific clade of relatively high diversity that contributed to the compartmentalization.

We recognize that cell-associated viral sequences derived from genital specimens could potentially include sequences from blood due to HIV-1 infected cells traversing through blood vessels in the cervix. To evaluate this possibility we assumed that if genital cell-associated sequences were actually from blood, their distribution within the phylogram should mirror blood. Visual examination of the phylograms reveal in most subjects that genital tract DNA sequences cluster in large part with other genital RNA or DNA sequences (Figure 1–4). Notably, viral clades composed only of cervical DNA sequences were observed in six subjects (Subjects 2, 4, 5, 8, 9 and 13, Figure 1–4); an unexpected pattern if HIV-1 DNA were derived from blood contamination. To further assess the likelihood that HIV-1 DNA genital sequences originated in genital tract, we evaluated the tissue origin of the nearest neighbor to each cell-associated genital sequence in the maximum likelihood phylograms of all 13 subjects. In this analysis the nearest neighbor to genital tract DNA sequences were other genital sequences in a median of 64% of the comparisons (range 30–93%) (Table 4). The observation that in all but one woman (Subject 3) we studied, the majority of genital DNA sequences group with other genital sequences suggests these likely originate from genital tissues, while the origin of cell-associated genital sequences that do not cluster with other genital viruses is less certain.

**Table 4:** The proportion of the genital biopsy sequences with the nearest neighbor sequence originating from the genital tract.

Treatment Status	Subject ID	Actual proportion of nearest neighbor from genital tract^a^	95% Confidence Interval	Expected proportion of nearest neighbor from genital tract^a^
“Effective” ART	1	71.43	43.95, 88.85	47.50
	2	61.54	34.36, 83.02	35.29
“Failing” ART	3	30.39	11.52, 59.42	16.18
	4	63.64	33.87, 85.67	24.62
	5	92.98	73.55, 98.44	35.71
	6	60.00	29.74, 84.17	45.65
No ART	7	70.59	45.81, 87.20	28.57
	8	63.93	29.68, 88.16	20.83
	9	93.34	76.46, 98.37	32.43
	10	70.00	37.63, 90.02	19.57
	11	53.41	45.62, 61.04	35.00
	12	55.55	25.13, 82.32	32.84
	13	60.00	29.74, 84.17	23.08

a Pair-wise distance were used to identify whether the nearest neighbor to each genital DNA sequence was another genital sequence or from blood, as described in materials and methods. The actual proportion indicates the proportion of genital DNA sequences that had a nearest neighbor that is also from the genital tract; generalized estimating equations (GEE) was used to obtain confidence intervals for the actual proportion. The expected proportion indicates the proportion of sequences that would be matched with a genital tract sequence, under the assumption of random matching.

### Amino acid sequence signatures and non-synonymous/synonymous (dN/dS) site changes

No signature amino acid residues were identified in HIV-1 sequences from the genital tract or peripheral blood of the 13 participants (data not shown). Sites undergoing positive selection were found in all 13 subjects. However, none were associated with compartmentalization of virus between the blood and genital tract (Supplemental Table S1), nor were there significant differences in the number of positively selected sites between genital tract and blood (p = 0.48 Wilcoxon Rank Sum Test).

### N-linked glycosylation and compartmental structure

The number of potential N-linked glycosylation sites per sequence was significantly greater (Kruskal Wallis test p<0.05) in the blood of three (2, 3, and 9) and in the genital tract of two women (8, 10; data not shown). Overall, no trend was noted for N-linked glycosylation sites in genital compared to blood viruses (p = 0.59 Wilcoxon Rank Sum Test).

### Predicted co-receptor usage

CCR5 was predicted to be the predominant co-receptor for virus in all of the subjects studied. HIV-1 viruses from three women were also predicted to use the CXCR4 co-receptor, including viruses from both the genital tract and peripheral blood. The CCR5- and CXCR4-using viruses clustered separately in phylogenetic trees (Subjects 2, 4 and 12, Figure 1 and 3); but were not restricted to the blood or genital tract and therefore did not appear to be compartmentalized.

## Discussion

HIV-1 *env* sequences were compartmentalized between the genital tract and blood in half the women by two or more statistical analyses. However, subjects' HIV-1 was rarely classified as compartmentalized by all four analyses, as indicated by moderate concordance values. Our findings demonstrate that the statistical algorithm used to evaluate the distribution of tissue-specific sequences within a phylogram affects the outcome., consistent with a previous study comparing statistical methods to evaluate compartmentalization of HIV-1 between the female genital tract and blood [35]. Of note, visual examination of the phylograms revealed clusters of monotypic and low-diversity variants interspersed across the phylograms of all subjects with compartmentalization by two or more tests. These tissue-specific monotypic and low diversity clusters impacted the statistical tests as shown by analyses conducted after collapsing monotypic variants into a single sequence in most if not all the subjects.

Phylogenetic analyses found tissue-specific clades with strong bootstrap support in half of the participants. However, in all but two women small clades of viruses comprised of blood or genital tract sequences intermingled in the phylograms. Across the subjects, each genital clade with >70% bootstrap support includes viruses with little genetic diversity suggestive of a burst of viral replication similar to that observed for viruses from macaque cervices [45] and human spleens [46], [47]. Given that the diameter of cervical specimens measured ∼3mm, and that sequential tissue sections were cut with a microtome for analysis, it is possible that the HIV-1 DNA sequences derived from these tissues came from adjacent or nearby cells. Genital secretions were similarly collected only from one region of the cervical *os*. Both sampling strategies could result in clades of monotypic or low diversity variants, derived by single-genome sequencing, due to sampling limited regions of the female genital tract where viruses are replicating. However, monotypic viruses may not be solely due to replicating viruses within a discrete area of tissue. When we biopsied multiple regions of the cervix monotypic viruses were observed across the cervix, and to a lesser extent in the blood [24]. Similarly, others' have detected monotypic and low diversity HIV-1 *env* from cervicovaginal lavages (CVL), which should sample the entire cervical and vaginal surfaces, and presumably represent the most fit variant [11], [15], [18], [48]. Our detection of monotypic variants in PBMC and blood plasma, which mix as the blood circulates, suggests the possibility of a systemic phenomenon. We hypothesize that monotypic viral variants may be from clonal expansion of HIV-1 infected cells, that when activated produce virions with identical genomes, as we [24], [27] and others [7], [49] have previously observed.

Further support for mixing of genital and blood HIV-1 comes from additional comparisons of the HIV-1 *env* populations from uterine cervix and blood. Across our subjects we did not observe tissue-specific signature amino acid residues, differential co-receptor usage, or evidence for differential immune selection (as measured by the patterns of non-synonymous site mutations) in either genital tract or blood viruses. Viral population entropy and diversity are also comparable in the genital tract and peripheral blood of subjects we and others [10], [15] have studied, which suggests similar immune pressures or mixing of viruses between these two tissues. The detection of viruses predicted to use CXCR4 (X4) co-receptor in both genital tract and blood viruses of our and others' subjects [15] suggests that X4 variants co-evolve or mix between the genital tract and blood, and importantly shows that similar to men [1], [50] X4 variants are not excluded from the genital tract of women.

Taken together, our data suggest that when statistical analyses of HIV-1 detect compartmentalization of genital tract and blood viruses in cross-sectional studies it is often due to low diversity or monotypic clusters, typical of a burst of replication or proliferation of a cell with provirus within the genital tract. The interspersed arrangement of genital and blood clades across the phylograms of most subjects we studied questions whether the genital population in each woman is distinct from viral population in her blood.

Across the literature, compartmentalization of HIV-1 within the genital tract of women by statistical measures and detection of discordant patterns of drug-resistance has been interpreted as evolution of independent viral populations within the genital tract [7], [9], [11], [12], [13], [15], [17]. Often these data were from cross-sectional studies of cell-free viruses obtained from plasma and CVL from women with ongoing viral replication, which suggests that unique variants could predominant in the tissues for a period of time [8], [9], [11], [12], [13], [15], [16], [17], [48].

Previous studies eliminated identical sequences from the analysis [7], [17] or evaluated only sequences with ≥0.3% genetic diversity [11] and still found marked compartmentalization. Nevertheless, the HIV-1 *env* sequences analyzed were often low diversity (<1%) cell-free virus that is consistent with recent rounds of viral replication [11], [15], which we contend biases analyses of compartmental structure. Longitudinal analyses corroborate our contention that sampling sites of replicating viruses or proliferating cells with provirus may bias towards compartmentalization. An analysis that includes sequences from specimens collected over time beginning with primary infection from three women, found the HIV-1 *env* sequences clustered by the sampling time as well as tissue type [7]. Therefore, statistical models used to compare HIV-1 populations between tissues may need to account for the effects of viruses from recent replication or proliferation of cells containing HIV-1 provirus.

Two longitudinal studies of drug resistance mutations in genital tract secretions and blood detected mutational discordance from one study visit that resolved at the next study visit [12], [51]. While these observations support mixing of viruses between tissues over time, we acknowledge that mutants could be selected in parallel. Others have noted that sequences from replicating viruses can predominate and obscure previously selected viral variants [7], [48], [52], raising the question whether the momentary snapshot of virus populations in cross-sectional analyses persists over time.

A potential difference between our study and those focused on cell-free virions centers on the observation that approximately 33% of 311 women [8] do not shed HIV-1 from the genital tract when virions are detectable in the plasma. In our study genital cell-associated viruses were characterized even in the absence of detectable cell-free virus. The origin of virions shed from the genital tract is unclear. Potentially, the virus shed into the vagina may be derived from a subset of resident genital cells or come from lymphocytes and/or macrophages that migrate into the genital tract in response to an infectious or other antigenic stimulus. Our observation that HIV-1 RNA sequences from the cervical secretions (RNA amplified from 8 of 13 subjects) clustered with sequences amplified from the cervical DNA supports the hypothesis that genital tract virions come from cells that were sampled in genital biopsy tissue. Consistent with this phylogenic clustering of genital RNA and DNA is an increase in tissue-specific compartmentalization (data not shown). However, genital virions may also have resulted from plasma transudates or bleeding, as a few genital RNA sequences cluster with plasma sequences in phylograms.

A limitation of our study is the uncertainty that genital HIV-1 DNA and RNA indeed originate from the genital tract, and are not due to blood contamination of the cervical biopsies or Sno-strip samples. Several experimental controls and analyses suggest that the majority of sequences we derived from the genital tract were likely from *in situ* viruses and not blood contaminates similar to others' observations [10], [12], [53]. First, blood contamination of Sno-strips (slightly pink tinged) with cervical secretions was noted in only 3/13 subjects, including Subject 9 who had compartmentalization of genital tract viruses in three of the four statistical tests and the least “mixing” between blood and genital sequences in the phylograms across all the study participants. Others have reported that genital viral loads tend to be stable over the menstrual cycle and that blood contamination contributes <1% of the genital tract viral load [9], [22]; further suggesting that pink tinged Sno-strip samples are not substantially contaminated with HIV-1 from the blood. Second, the absence of template amplification in our “no reverse transcriptase controls” performed using nucleic acids extracted from genital secretions, suggests that HIV-1 DNA does not appreciably contaminate Sno-strip specimens and masquerade as viral RNA. Third, while we cannot definitely exclude the possibility that viral sequences derived from DNA extracts of cervical tissue are free of HIV-1-infected blood cells traversing through the tissue, two observations indicate that sequences derived from genital DNA likely originated from viral replication in the genital tract: Phylogenetic clustering of genital sequences have strong bootstrap support for viral clades composed only of cervical DNA sequences; and the nearest neighbor sequence to the majority of genital DNA is another genital tract sequence even in instances where blood sequences outnumbered genital sequences.

In summary, our analyses of viral sequences from the blood and genital tract of women with chronic HIV-1 infection suggest that the multiple clusters of genetically similar and monotypic viruses, particularly from the cervix, are likely from recent viral replication or proliferation of cells with provirus, respectively. Statistical tests used to assess virus compartmentalization appear biased by these tissue-specific low-diversity viruses over the intermingling of blood and genital clades evident in phylograms. If our hypothesis is correct, then few if any significant barriers exist to the flow of HIV-1 between the female genital tract and blood, and unique genital tract viral lineages should not evolve and persist over time, which is a hallmark of a viral compartment [54]. Longitudinal studies are warranted to further define HIV-1 evolution in the female genital tract; and to further investigate whether the perception that female genital tract HIV-1 evolves separately from blood is mistaken.

## Supporting Information

Table S1 Sites with Positive Selection and Entropy Differences
(0.43 MB TIF)
