# Supplementary figures and images for: Compartmentalization of HIV-1 within the Female Genital Tract Is Due to Monotypic and Low-Diversity Variants Not Distinct Viral Populations
Source: PLoS One. 2009 Sep 22;4(9):e7122. doi: 10.1371/journal.pone.0007122 (PMC2741601; doi:10.1371/journal.pone.0007122)

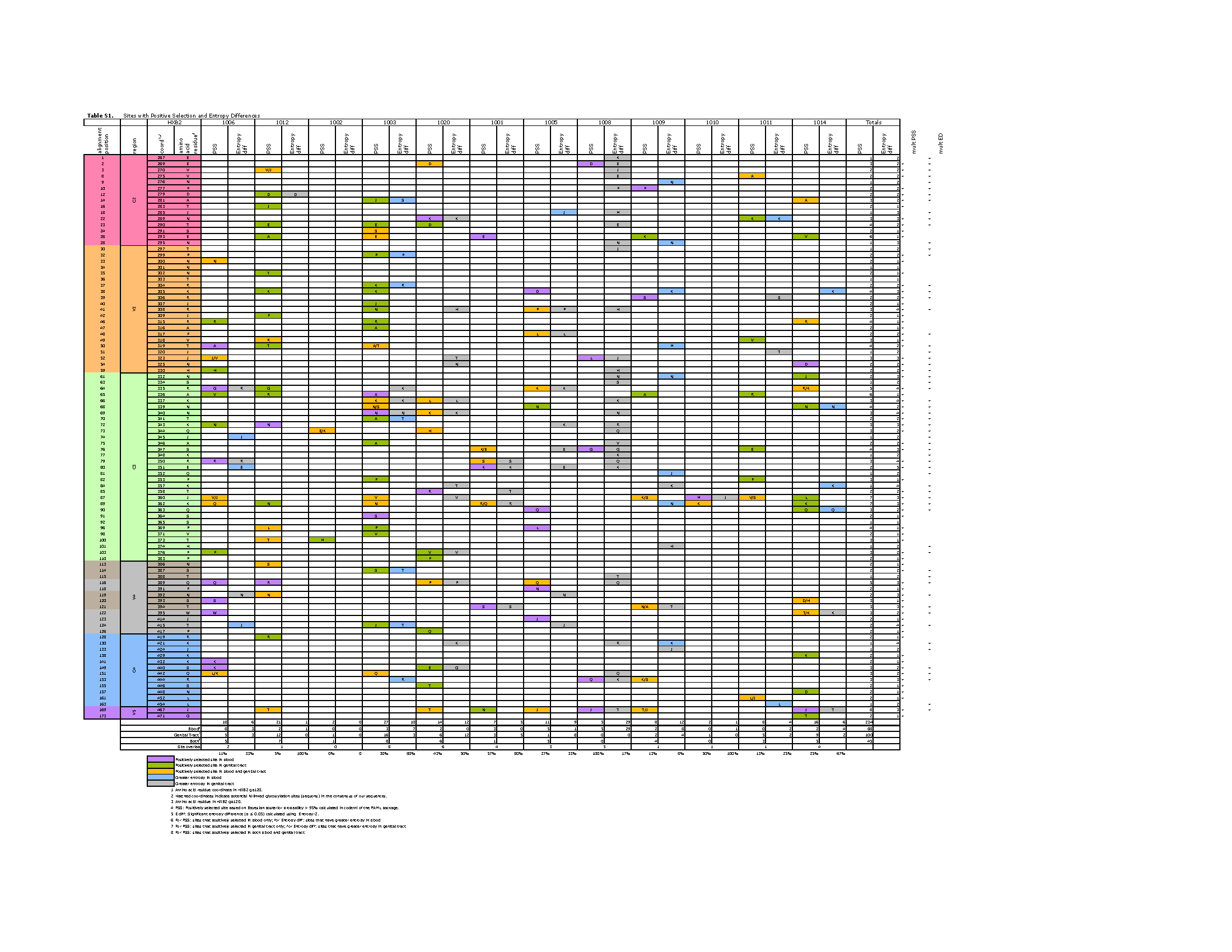

Supplement: Table S1 — Sites with Positive Selection and Entropy Differences (0.43 MB TIF) [file pone.0007122.s001.tif]
